# NLRP3-dependent microglial training impaired the clearance of amyloid-beta and aggravated the cognitive decline in Alzheimer’s disease

**DOI:** 10.1038/s41419-020-03072-x

**Published:** 2020-10-13

**Authors:** Xiao-fei He, Jing-hui Xu, Ge Li, Ming-yue Li, Li-li Li, Zhong Pei, Li-ying Zhang, Xi-quan Hu

**Affiliations:** 1 Department of Rehabilitation Medicine, The Third Affiliated Hospital, Sun Yat-sen University, 510630 Guangzhou, Guangdong China; 2 The Eighth Affiliated Hospital, Sun Yat-sen University, 518000 Shenzhen, Guangdong China; 3 Guangdong Provincial Key Laboratory of Laboratory Animals, Guangdong Laboratory Animals Monitoring Institute, 510663 Guangzhou, Guangdong China; 4 Department of Neurology, National Key Clinical Department and Key Discipline of Neurology, Guangdong Key Laboratory for Diagnosis and Treatment of Major Neurological Diseases, The First Affiliated Hospital, Sun Yat-sen University, 510080 Guangzhou, Guangdong China

**Keywords:** Cellular neuroscience, Alzheimer's disease, Inflammasome

## Abstract

Alzheimer’s disease (AD), the most common form of dementia, is marked by progressive cognitive decline, deposition of misfolded amyloid-β (Aβ) peptide and formation of neurofibrillary tangles. Recently, microglial training has emerged as an important contributor to neurological diseases, which augments the subsequent inflammation. However, how it affects the pathology of AD remains unknown. Here, using a mouse model of sporadic Alzheimer’s disease (SAD) induced by streptozotocin injection, we demonstrated that microglial training exacerbated Aβ accumulation, neuronal loss, and cognitive impairment. In addition, we injected MCC950 to inhibit NLRP3 activation and used an inducible Cre recombinase to delete the NLRP3 gene in microglia. Inhibition or depletion of microglial NLRP3 could protect against the pathologies of SAD and abolish the effects of microglial training. Our results identified microglial training as an important modifier of neuropathology in SAD and demonstrated that activation of NLRP3 inflammasome contributed to the pathologies and microglial training in SAD. Therefore, NLRP3 could be a potential therapeutic target for SAD treatment.

## Introduction

Alzheimer’s disease (AD) is characterized by amyloid-beta (Aβ) deposition and neurofibrillary tangle formation in brain^1^. Senile plaques activate microglia and drive cerebral neuroinflammation^2^, neuroinflammation has been termed as the third core pathological feature in AD^3^. Aβ deposition precedes the development of cognitive deficits in AD by several years^4^, it is therefore important to understand the regulation of microglial response to prevent or delay the pathological process of AD.

Microglia is related to phagocytosis of Aβ^5^, elimination of synapse^6^ and immune training^7,8^. Trained microglia responds in an enhanced manner to subsequently unspecific stimuli^9^. Activation of the NACHT-, LRR-, and pyrin (PYD)-domain-containing protein 3 (NLRP3) inflammasome contributes to microglial phenotype skewing and Aβ deposition in AD^10^. Recently, NLRP3 has also been reported to mediate the immune training in myeloid cells^11^. However, it has not been investigated whether NLRP3 regulates microglial training in AD. There is an urgent need to explore the regulation of microglial training, because it exacerbates AD pathology by remembering peripheral inflammation for years.

Streptozotocin (STZ) induces many pathological changes associated with sporadic Alzheimer’s disease (SAD)^12^. In present study, combining a mouse model of SAD and subsequently systemic inflammation, we investigated the role of NLRP3 inflammasome in the pathological process of SAD as well as in microglial training. Our results indicated that injection of STZ activated the NLRP3 inflammasome, impaired the microglial and glymphatic clearance of Aβ and eventually induced neuronal loss and cognitive impairment. Peripheral inflammation enhanced the activation of NLRP3, amplified the microglial response, and exacerbated the pathological damage in SAD. Inhibition or depletion of microglial NLRP3 restricted the neuroinflammation, improved the microglial and glymphatic clearance of Aβ, protected against the neuronal loss and improved the cognitive function.

## Results

### Peripheral inflammation aggravated the neurological dysfunction in SAD in a NLRP3-dependent manner

**Fig. 1:**
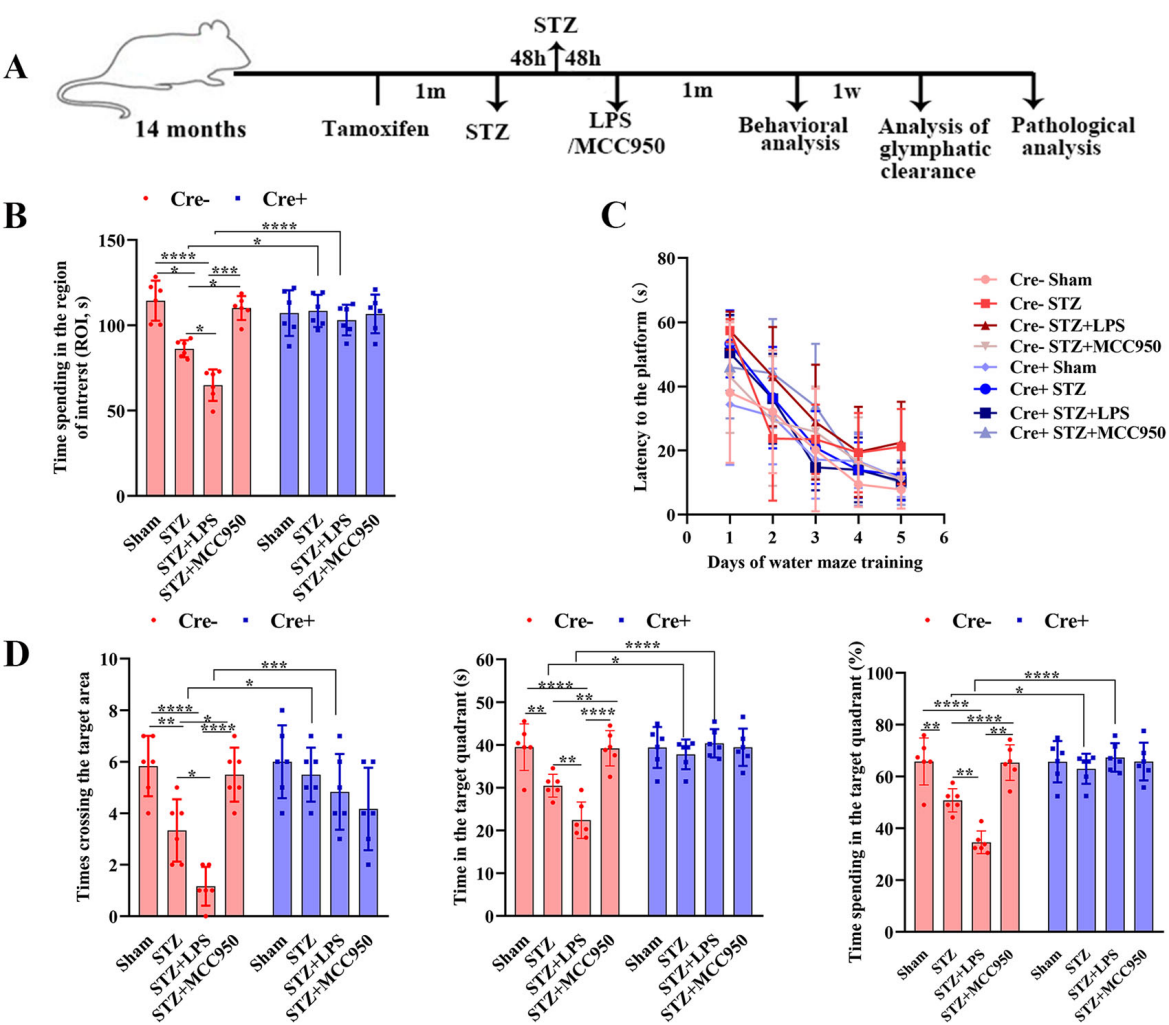
Schematic of the experimental timeline and analysis of behavioral test. **A** Time point of the schematic design. **B** Analysis of time spending in the central area (Region of interest, ROI) in the open field test. **C** Analysis of latencies to the platform during the training days of the Morris water maze task. **D** Comparisons of the times crossing the target area and time spent in the target quadrant during the probe trial. Each dataset is expressed as mean ± SD. ^*^P ≤ 0.05; ^**^P ≤ 0.01; ^***^P ≤ 0.001; ^****^P ≤ 0.0001. *n* = 6 mice.

In open field test, time spent in the region of interest (ROI) was significantly decreased in STZ group in Cre− mice compared with that in sham group, it was further decreased in STZ + lipopolysaccharide (LPS) group, whereas increased in STZ + MCC950 group (Fig. 1B). Time spent in the ROI in STZ and STZ + LPS groups was significantly increased in Cre+ mice compared with that in Cre− mice. These results indicated that STZ injection caused anxiety, which was aggravated by peripheral inflammation but attenuated by inhibition or depletion of microglial NLRP3.

In Morris water maze test, latencies to the platform on day 5 were significantly increased in the STZ and STZ + LPS groups in Cre− mice compared with those in sham groups. They were also significantly increased compared with those in STZ and STZ groups in Cre+ mice (Fig. 1C). During the probe trial (Fig. 1D), the number of times crossing the former platform site was significantly decreased in STZ group compared with that in sham group in Cre− mice, which was further decreased in STZ + LPS group whereas increased in STZ + MCC950 group (Fig. 1D). However, these numbers in STZ and STZ + LPS groups were significantly increased in Cre+ mice compared with those in Cre− mice. In Cre− mice (Fig. 1D), the time spent in the target quadrant was significantly decreased in STZ group compared with that in sham group; it was further decreased in STZ + LPS group but increased in STZ + MCC950 group, the time spent in the target quadrant was significantly increased in STZ and STZ + LPS groups in Cre+ mice compared with those in Cre− mice. These results indicated that STZ injection impaired the spatial reference learning and memory, which were exacerbated by peripheral inflammation but attenuated by inhibition or depletion of microglial NLRP3.

### Peripheral inflammation aggravated the activation of microglial NLRP3

**Fig. 2:**
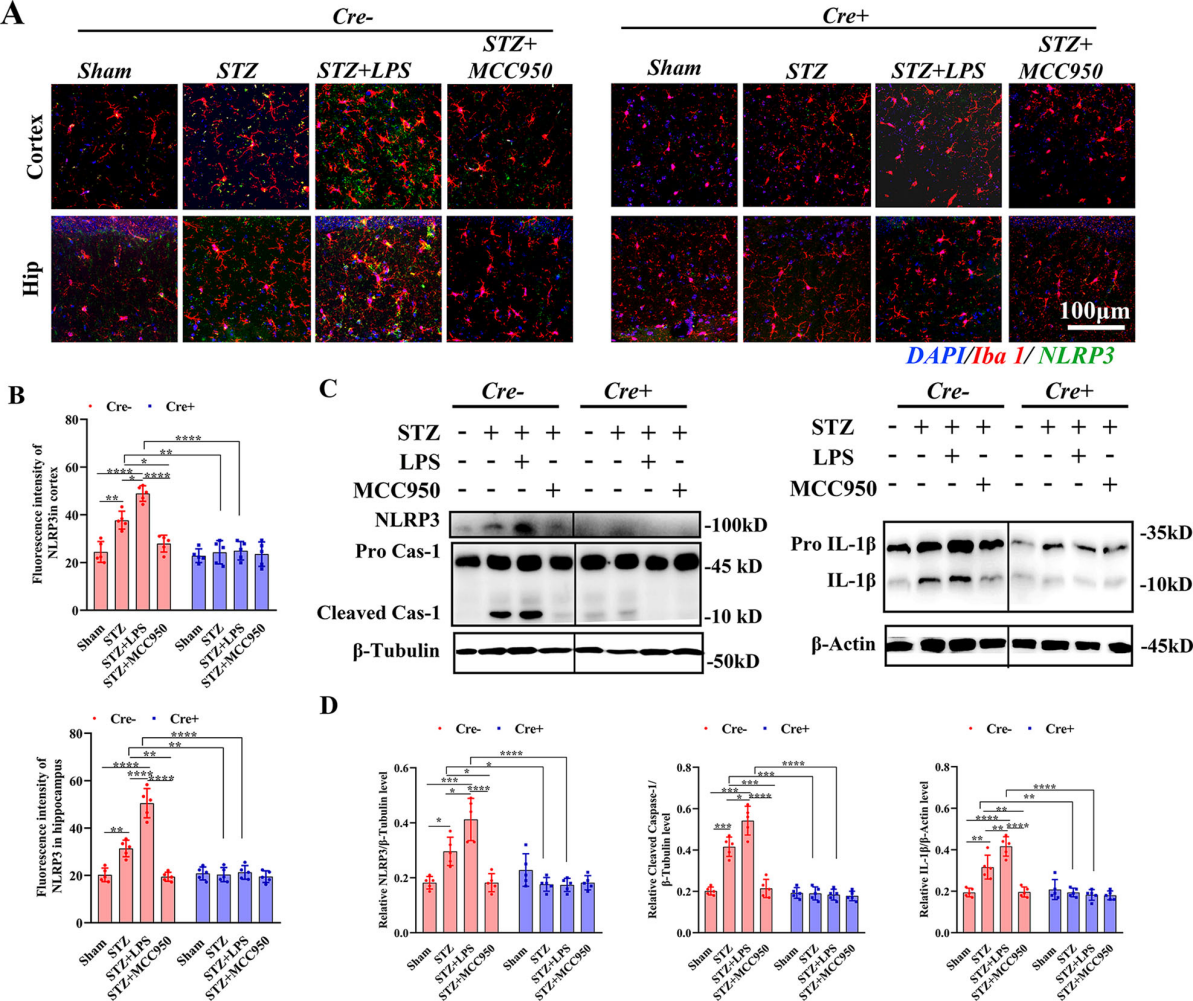
Histological and western blotting analysis of NACHT-, LRR-, and pyrin (PYD)-domain-containing protein 3 (NLRP3) activation. **A** Immunofluorescence staining of NLRP3 and ionized calcium-binding adapter molecule 1 (Iba1) (×63 oil immersion objective). **B** Comparisons of NLRP3 intensities in the cortex and hippocampus. **C** Chemiluminescence images of NLRP3, caspase-1 and β-tubulin, IL-1β and β-actin. **D** Comparisons of the NLRP3/β-tubulin, cleaved caspase-1/β-tubulin, and IL-1β/β-actin ratios. Each dataset is expressed as mean ± SD. ^*^P ≤ 0.05; ^**^P ≤ 0.01; ^***^P ≤ 0.001; ^****^P ≤ 0.0001. *n* = 6 mice.

In Cre− mice, the mean fluorescence intensities of NLRP3 in the cortex and hippocampus were significantly increased in STZ group compared with those in sham group, which were further increased in STZ + LPS group whereas decreased in STZ + MCC950 group (Fig. 2A and B), the intensities in STZ and STZ groups were significantly decreased in Cre+ mice compared with those in Cre− mice. Three-dimensional analysis showed that the NLRP3 inflammasomes in Cre− mice were mostly located in the microglia (Supplementary Fig. 1A and B). Western blotting analysis showed that in Cre− mice, the levels of NLRP3, cleaved caspase-1 and IL-1β were significantly increased in STZ group compared with those in sham group (Fig. 2C and D), LPS injection further increased whereas MCC950 decreased the levels of NLRP3, cleaved caspase-1, and IL-1β. There was no obvious NLRP3 expression in Cre+ mice, the levels of cleaved caspase-1, and IL-1β were significantly decreased in STZ and STZ + LPS groups in Cre+ mice compared with those in Cre− mice. These results indicated that NLRP3 inflammasomes in brain were mainly located in microglia, which could be abolished by TAM administration in Cre+ mice. STZ administration activated the NLRP3 inflammasome, which was exacerbated by peripheral LPS injection but inhibited by MCC950 administration.

### NLRP3 inflammasome was required for microglial training in SAD

**Fig. 3:**
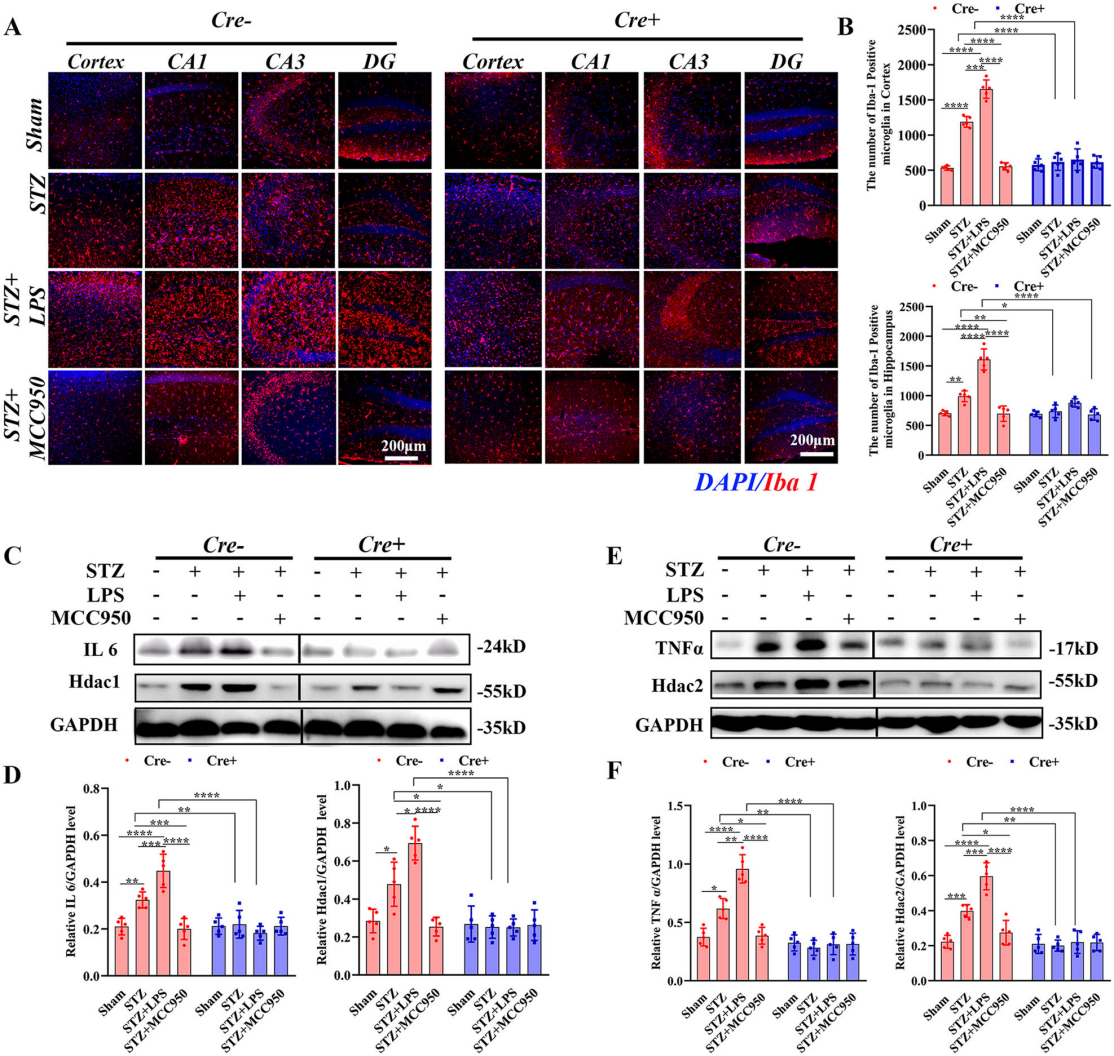
Histological and western blotting analysis of microglial activation, pro-inflammatory cytokines, and histone deacetylase (Hdac)1/2 levels. **A** Immunofluorescence staining of Iba1 in the cortex and hippocampus. **B** Comparisons of the numbers of Iba1-positive microglia in the cortex and hippocampus (×40 objective). **C** Chemiluminescence images of IL-6, Hdac1, and GAPDH. **D** Comparisons of the IL-6/GAPDH, Hdac1/GAPDH ratios. **E** Chemiluminescence images of TNF-α, Hdac2, and GAPDH. **F** Comparisons of the TNF-α/GAPDH, Hdac2/GAPDH ratios. Each dataset is expressed as mean ± SD. ^*^P ≤ 0.05; ^**^P ≤ 0.01; ^***^P ≤ 0.001; ^****^P ≤ 0.0001. *n* = 6 mice.

To evaluate the involvement of NLRP3 inflammasome in microglial training, mice received one low-dose of LPS or MCC950 injection after STZ administration. As shown in Fig. 3, in Cre− mice, the numbers of microglia in the cortex and hippocampus (Fig. 3A and B) were significantly increased in STZ group compared with those in sham group, which were further increased in STZ + LPS group but decreased in STZ + MCC950 group. These numbers of microglia in the cortex and hippocampus were significantly decreased in STZ and STZ + LPS groups for Cre+ mice compared with those in Cre− mice.

In Cre− mice, levels of IL-6 (Fig. 3C and D) and TNF-α (Fig. 3E and F) were significantly increased in STZ group compared with those in sham group, which were further increased in STZ + LPS group but decreased in STZ + MCC950 group. They were significantly decreased in STZ and STZ + LPS groups in Cre+ mice compared with those in Cre− mice. STZ injection increased the levels of histone deacetylase 1/2 (Hdac1/2) compared with those in sham group in Cre− mice. LPS further increased the levels of Hdac1/2 whereas MCC950 decreased the level of Hdac1. Besides, levels of Hdac1/2 in STZ and STZ + LPS groups were significantly decreased in Cre+ mice compared with those in Cre− mice.

To further elucidate the involvement of NLRP3 inflammasome in microglial training, mice received daily injection of low-dose LPS on two consecutive days. In Cre− mice, 1×LPS led to significant increase of Hdac1/2, TNF-α, and IL-6 compared with control group. The increase was more dramatic with the 2×LPS injections (Fig. S2A–D). Comparing with the control group, 1×LPS increased the levels of NLRP3, cleaved caspase-1, IL-1β in Cre− mice, which were further increased by 2×LPS injections (Fig. S2E–H). There was no obvious expression of NLRP3 inflammasomes in Cre+ mice, and there were no significant effects of 1×LPS or 2×LPS injections on cleaved caspase-1, IL-1β. These results indicated that peripheral inflammation induced the effects of immune training, which were abolished by inhibition or depletion of microglial NLRP3.

### Peripheral inflammation aggravated the Aβ burden in a NLRP3-dependent manner

**Fig. 4:**
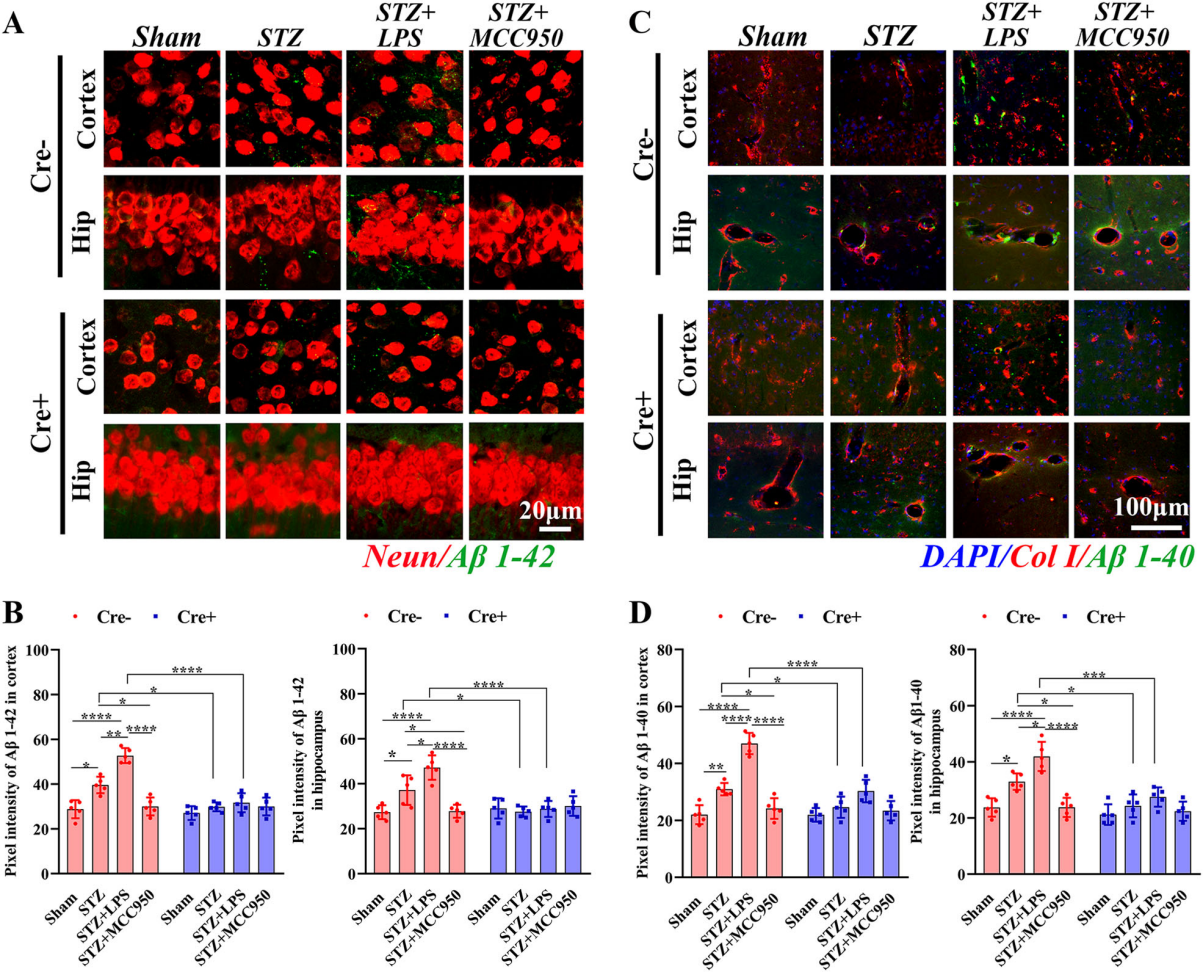
Histological analysis of Aβ deposits. **A** Immunofluorescence staining of Neurons and Aβ 1-42 fragments (×63 oil immersion objective, zoomed in 3). **B** Comparisons of Aβ 1-42 fragments in the cortex and hippocampus. **C** Immunofluorescence staining of Aβ 1-40 fragments and collagen I (Col I) (×60 objective). **D** Comparisons of Aβ 1-40 fragments in the cortex and hippocampus. Each dataset is expressed as mean ± SD. ^*^P ≤ 0.05; ^**^P ≤ 0.01; ^***^P ≤ 0.001; ^****^P ≤ 0.0001. *n* = 6 mice.

To examine the effect of microglial training on the Aβ deposition as well as the involvement of microglial NLRP3, we performed immunofluorescence staining of Aβ. In Cre− mice, the mean fluorescence intensities of Aβ1-42 (Fig. 4A, B) and Aβ1-40 (Fig. 4C, D) fragments in cortex and hippocampus were significantly increased in STZ group compared with those in sham group. LPS injection further increased whereas MCC950 decreased the Aβ1-42 and Aβ1-40 deposits, they were significantly decreased in STZ and STZ + LPS groups in Cre+ mice compared with those in Cre− mice. Our results indicated that STZ-induced Aβ deposition, which was exacerbated by peripheral inflammation but attenuated by inhibition or depletion of microglial NLRP3.

### Peripheral inflammation exacerbated the dysfunction of microglial clearance in a NLRP3-dependent manner

**Fig. 5:**
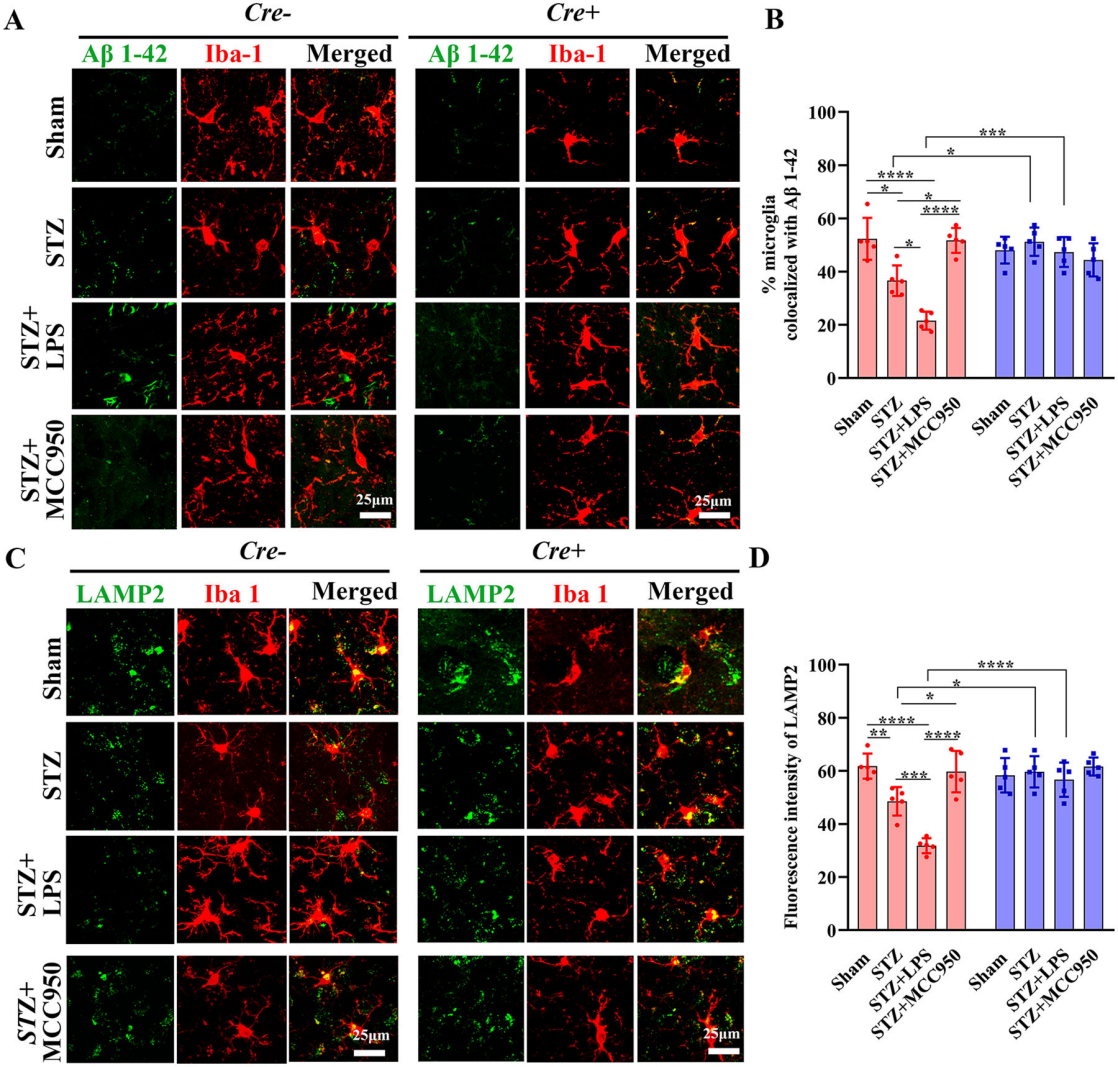
Histological analysis of microglial Aβ phagocytosis. **A** Representative confocal images showing co-localization of Iba1+ microglia (red) with Aβ 1-42 fragments (green) in the hippocampus (×63 oil immersion objective, zoomed in 3). **B** Comparisons of % (n/n) microglia that co-localized with Aβ 1-42 fragments. **C** Representative images showing the co-localization of Iba1+ microglia (red) with LAMP2 (green) (×63 oil immersion objective, zoomed in 3). **D** Comparisons of fluorescence intensities of LAMP2. Each dataset is expressed as mean ± SD.^*^P ≤ 0.05; ^**^P ≤ 0.01; ^***^P ≤ 0.001; ^****^P ≤ 0.0001. *n* = 6 mice.

We quantified the Iba1+ microglia that were co-localized with Aβ 1-42 fragments to investigate the microglial phagocytosis^13^. In Cre− mice, the percentage of microglia that co-localized with Aβ 1-42 was significantly decreased in STZ group compared with that in sham group, which was further decreased in STZ + LPS group but increased in STZ + MCC950 group. Besides, the percentages in STZ and STZ + LPS groups were significantly increased in Cre+ mice compared with those in Cre− mice (Fig. 5A and B). The lysosomal-associated membrane protein 2 (LAMP2) is termed as activated phagocytic microglia^14^. In Cre− mice, the mean fluorescence intensity of LAMP2 was significantly decreased in STZ group compared with that in sham group; LPS injection further decreased whereas MCC950 increased the LAMP2 intensity (Fig. 5C and D). The LAMP2 intensities in STZ and STZ + LPS groups were increased in Cre+ mice compared with those in Cre− mice. Our results suggested that peripheral inflammation inhibited the phagocytic event in microglia, which could be improved by inhibition or depletion of microglial NLRP3.

### Peripheral inflammation aggravated the astrocytic dysfunction in SAD in a NLRP3-dependent manner

**Fig. 6:**
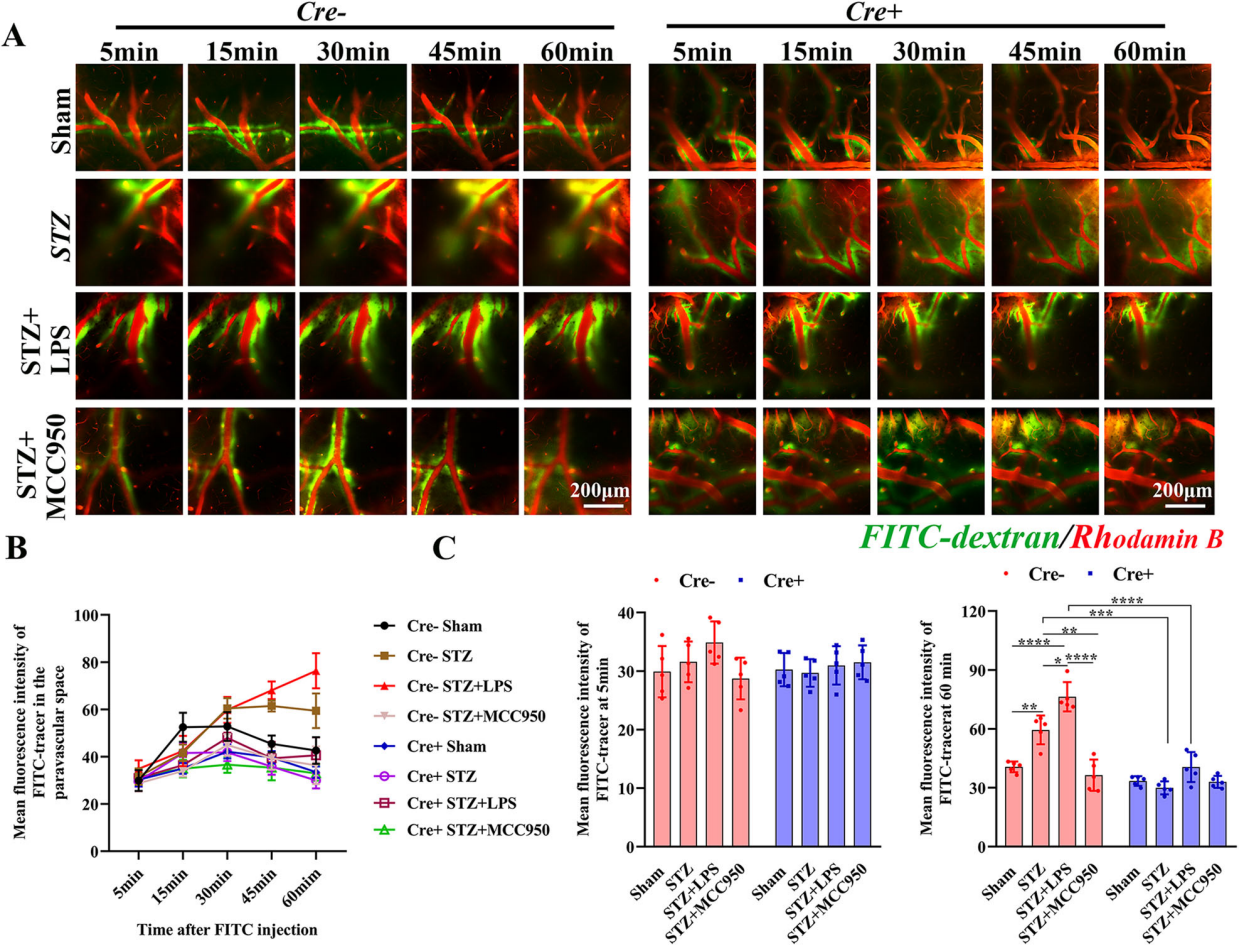
In vivo two-photon imaging analysis of glymphatic clearance. **A** Representative two-photon images showing the change about fluorescence intensities of FITC at different time after injecting into cisterna magna, indicating the clearance of FITC-dextran in glymphatic pathway (×25 water immersion objective). **B** Linear analysis of the fluorescence intensities of FITC-dextran at 5, 15, 30, 45, and 60 min after injection. **C** Histogrammic analysis of the fluorescence intensities of FITC-dextran at 5 and 60 min after injection. Each dataset is expressed as mean ± SD. ^*^P ≤ 0.05; ^**^P ≤ 0.01; ^***^P ≤ 0.001; ^****^P ≤ 0.0001. *n* = 6 mice.

Neuroinflammation has been reported to impair the glymphatic function and induce Aβ accumulation^15^. To examine the glymphatic function in SAD as well as the regulation of microglial training, we detected the glymphatic function using in vivo two-photon imaging (Fig. 6). After injection of FITC-dextran, the fluorescence intensities kept increasing in STZ and STZ + LPS groups in Cre− mice, but they reached peak at 15 or 30 min in other groups, after which they decreased at 45 and 60 min (Fig. 6A and B). Specifically, the FITC intensities at 5 min showed no significant differences among these four groups both for Cre− and Cre+ mice. But at 60 min after injection, the fluorescence intensity was significantly increased in STZ group compared with that in sham group, which was further increased in STZ + LPS group but decreased in STZ + MCC950 group in Cre− mice. They were significantly decreased in STZ and STZ + LPS groups for Cre+ mice compared with those in Cre− mice (Fig. 6C).

Activated microglia induces the A1 reactive astrocytes^16^, and reactive astrocytes directly lead to a loss of aquaporin4 (AQP4) polarization from the end-feet to the soma, which impairs the glymphatic function^17^. Therefore, we examined the activation of astrocyte. In Cre− mice, the fluorescence intensity of GFAP was increased in STZ group compared with that in sham group, both for cortex (Fig. S3A and B) and hippocampus (Fig. S3C and D), which was further increased in STZ + LPS group but decreased in STZ + MCC950 group. We then examined the function of astrocytic AQP4, the fluorescence intensities of AQP4 showed no significant differences among these four groups both for Cre− and Cre+ mice. However, in Cre− mice, the AQP4 polarity^18^ was decreased in STZ group compared with that in sham group, both for the cortex and hippocampus, which was further decreased in STZ + LPS group but increased in STZ + MCC950 group. The AQP4 polarities were significantly increased in STZ and STZ + LPS groups in Cre+ mice compared with those in the Cre− mice. As for the phenotype of astrocyte, in Cre− mice, the number of A1 astrocytes (C3-positive) was significantly increased in STZ group compared with those in sham group, both for cortex (Fig. S4A and B) and hippocampus (Fig. S4C and D), they were further increased in STZ + LPS group but decreased in STZ + MCC950 group. The numbers of A1 astrocytes in STZ and STZ + LPS groups were significantly decreased in Cre+ mice compared with those in Cre− mice.

### Peripheral inflammation aggravated the neuronal loss in SAD in a NLRP3-dependent manner

**Fig. 7:**
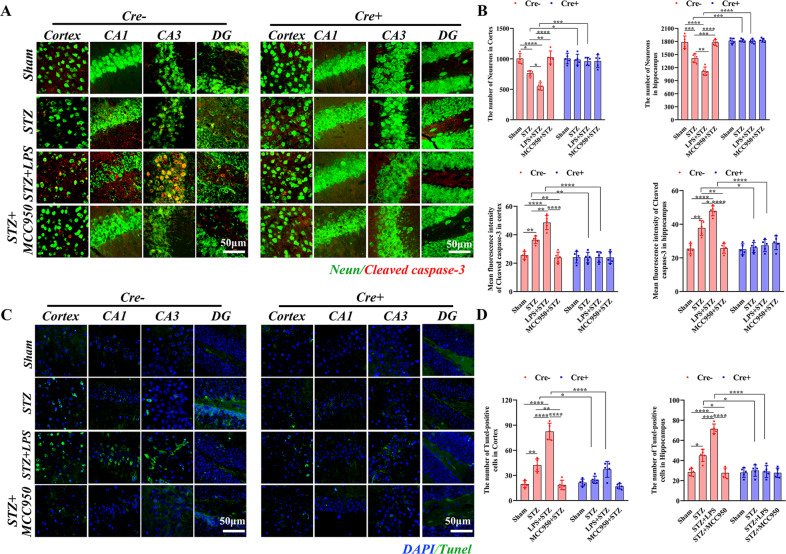
Histological analysis of neurons, cleaved caspase-3, and TUNEL-positive cells. **A** Representative images of Neurons and cleaved caspase-3 in Cre− and Cre+ mice (×63 oil immersion objective). **B** Comparisons of the numbers of neurons and the fluorescence intensities of cleaved caspase-3 in the cortex and hippocampus. **C** Representative confocal images of TUNEL-positive cells in the cortex and hippocampus (×63 oil immersion objective) in Cre− and Cre+ mice. **D** Comparisons of the numbers of TUNEL-positive cells in the cortex and hippocampus. Each dataset is expressed as mean ± SD. ^*^P ≤ 0.05; ^**^P ≤ 0.01; ^***^P ≤ 0.001; ^****^P ≤ 0.0001. *n* = 6 mice.

In Cre− mice, the number of neurons was significantly decreased in STZ group compared with those in sham group, both for cortex and hippocampus; LPS injection further decreased whereas MCC950 increased the numbers of neurons (Fig. 7A and B). Comparing between Cre− and Cre+ mice, the numbers of neurons in STZ and STZ + LPS groups were significantly increased in Cre+ mice both for the cortex and hippocampus. Besides, the fluorescence intensity of cleaved caspase-3 was significantly increased in STZ group compared with that in sham group both for the cortex and hippocampus (Fig. 7A and B), LPS further increased whereas MCC950 decreased the intensity of cleaved caspase-3. However, the expressions of cleaved caspase-3 were significantly decreased in STZ and STZ + LPS groups in Cre+ mice compared with those in Cre− mice. In Cre− mice, the numbers of TUNEL-positive cells was significantly increased in STZ group compared with that in sham group both for the cortex and hippocampus; LPS further increased whereas MCC950 decreased the TUNEL-positive cells (Fig. 7C and D). However, the TUNEL-positive cells were significantly decreased in STZ and STZ + LPS groups in Cre+ mice compared with those in Cre− mice, both for cortex and hippocampus. These results suggested that peripheral inflammation aggravated the neuronal loss induced by STZ injection, which could be attenuated by inhibition or depletion of microglial NLRP3.

## Discussion

Systemic inflammation increases the risk for developing dementia in AD^19^. Consistently, our study showed that systemic inflammation aggravated the Aβ deposition and cognitive impairment in the SAD model owing to microglial training. Indeed, STZ induced microglia into a pro-inflammatory state in Cre− mice, which was enhanced by subsequent injection of LPS, demonstrating that the microglia had been trained. Besides, our study demonstrated that NLRP3 inflammasome was critical for regulating the microglial training. Firstly, NLRP3 inflammasome was demonstrated to mediate trained immunity in myeloid cells^11^, we excluded the influence of myeloid cells in brain, because TAM depleted the NLRP3 in long lived microglia but not in *CX3CR1*^*CreER/+*^short-lived myeloid cells^20^. Secondly, LPS exacerbated the pro-inflammatory cytokines and Aβ deposition in Cre− mice, which was abolished in Cre+ mice. Thirdly, the increase of Hdac1/2 in Cre− mice might be considered as major regulator of epigenetic reprogramming of immune training in microglia^8^, they were decreased in Cre+ mice.

Microglial clearance is one of the critical pathways to remove the Aβ^21^, which was impaired in the STZ-induced SAD model; the impairment was enhanced by systemic inflammation but was improved by inhibition or depletion of microglial NLRP3. It is therefore supposed that NLRP3-mediated training exacerbated the impairment of microglial clearance of Aβ deposits. Firstly, systemic inflammation enhanced NLRP3 activation and increased the production of pro-inflammatory cytokines, they might decrease the Aβ-binding scavenger receptors and further reduced Aβ uptake^22^. Furthermore, LAMP2 is associated with phagocytosis and degradation of extra-cellular Aβ fibrils in microglia^14,23^, systemic inflammation exacerbated the decrease of LAMP2 in SAD mice. This may be due to the increased levels of Hdac1/2, because Hdac1/2 could inhibit the expression of LAMP2 in microglia and worsen the Aβ plaque burden^24^.

NLRP3-regulated microglial training exacerbated the dysfunction of the glymphatic pathway, which might also contribute to the Aβ deposition^25^. Firstly, reactive astrocytes directly lead to the loss of AQP4 polarization and impair the glymphatic function^17,18^. We found that the toxic A1 astrocytes were increased and the AQP4 polarities were decreased in SAD mice, which were aggravated by LPS injection in Cre− mice, but not in Cre+ mice. Secondly, activated microglia was reported to convert trophic astrocytes to toxic A1 astrocytes via pro-inflammatory cytokines^16^, we found the pro-inflammatory cytokines were decreased in Cre+ mice, which resulted in a decreased number of A1 astrocytes. Finally, astrocytic activation is delayed more than microglial training^8^. However, the glymphatic clearance was decreased and the A1 astrocytes were increased following STZ injection in Cre− mice in our study, which is different from the results reported by Wendeln et al.^8^, where the astrocytes were not activated until 3×LPS injection. This might because the age of mice we used were 14 months, the microglia may develop a primed profile and exhibit an exaggerated inflammatory response^26^. Finally, dysfunction of astrocytes also contributed to the increase of Aβ deposits, because dysfunctional astrocytes are unable to take up and degrade Aβ deposits^27^. It is worthy of note that NLRP3-regulated microglial training might also exacerbate the synaptic dysfunction in AD, because both activated microglia and A1 astrocytes induce synaptic loss^6,28^, inhibition of NLRP3 promotes the synaptic plasticity^29^.

In summary, our results demonstrated that activation of NLRP3 inflammasome plays a critical role in SAD. It impaired the microglial and glymphatic clearance, induced the Aβ deposition and neuronal loss and eventually resulted in cognitive decline. More importantly, NLRP3 inflammasome regulated the microglial training, it aggravated the pathological process of SAD. Inhibition or depletion of microglial NLRP3 protected against the pathologies of SAD as well as the deleterious effects of microglial training.

## Material and methods

### Animals

The study was approved by the Institutional Animal Care and Use Committee (IACUC) of Guangdong Laboratory Animal Monitoring Institute. *CX3CR1*^*CreER*^ mice were generated as described previously^30^, mice containing a floxed allele of *NLRP3* (*NLRP3*^*flox*^) were purchased from the Model Animal Research Center of Nanjing University (Stock Number: T000352). These mice were crossed to obtain *CX3CR1*^*CreER/+*^*:NLRP3*^*fl/fl*^ mice, they were bred at the Guangdong Laboratory Animals Monitoring Institute (Guangzhou, China). For deletion of NLRP3 (Cre+ mice), 8 mg of tamoxifen (TAM, Sigma, USA) was dissolved in corn oil, which was applied subcutaneously twice at an interval of 48 h^30^; corn oil without TAM was applied to the control mice (Cre−).

### Treatment schedule

Male mice were randomly divided into sham, STZ, STZ + LPS, and STZ + MCC950 groups. Sample size was estimated (*n* = 6) according to the previous articles^31,32^. Thirty days after TAM administration (Fig. 1A)^30^, mice in sham groups received two intracerebroventricular (ICV) infusions of citrate buffer; mice in STZ groups received bilateral ICV infusions of STZ at a dose of 3 mg/kg at interval of 48 h^33^; mice in STZ + LPS groups received STZ infusions followed by intraperitoneal injection of LPS (1 mg/kg, Sigma, USA); mice in STZ + MCC950 groups received STZ infusions followed by intraperitoneal administration of MCC950 (50 mg/kg, Sigma, USA)^34,35^. The experiment was designed in compliance with the ARRIVE guidelines and no exclusion of data was done. Thirty days after STZ administration, mice were subjected to behavioral test and vivo two-photon imaging analysis. Subsequently, the immunofluorescence staining and western blotting analysis were performed.

### In vivo two-photon imaging of glymphatic clearance

Mouse was anesthetized and a thin cranial window was created, fluorescein isothiocyanate (FITC)-dextran (Sigma, USA) was injected into the cisterna magna and 0.2 ml rhodamine B (Sigma, USA) was injected intravenously immediately before imaging. Two-photon imaging was performed as described elsewhere^15,18^, images were obtained at 5, 15, 30, 45, and 60 min after the injection of FITC-dextran.

### Western blotting analysis

Proteins were subjected to SDS–PAGE using 12.5% or 15% (v/v) precast polyacrylamide gels at 120 V for 90 min, they were transferred to polyvinylidene fluoride membranes (Millipore, USA) at 100 V for 2 h. Membranes were incubated in 5% (w/v) milk for 1 h and then with primary antibodies (mouse anti-NLRP3 antibody, Thermo Fisher, USA; rabbit anti-IL-1beta antibody, Abcam, USA; rabbit anti-caspase-1 antibody, Abcam, USA; rabbit anti-tubulin beta antibody, Affinity, USA; rabbit anti-beta actin antibody, Affinity, USA; rabbit anti-GAPDH antibody, Affinity, USA; rabbit anti-IL-6 antibody, Affinity, USA; rabbit anti-TNF-α antibody, Affinity, USA; rabbit Hdac1/2 antibodies, Affinity, USA) overnight at 4 °C. After washes, the membranes were incubated with related secondary antibodies for 1 h.

### Histology

Sections were boiled in citric acid buffer and were treated with 0.3% (v/v) Triton X-100 and 10% (v/v) goat serum, they were incubated overnight at 4 °C with primary antibodies (rabbit anti-ionized calcium-binding adapter molecule 1 (Iba1) antibody, Wako, Japan; mouse anti-NLRP3 antibody, Thermo Fisher, USA; mouse purified anti-β-amyloid, 1-42 antibody, BioLegend, USA; mouse purified anti-β-amyloid, 1-40 antibody, BioLegend, USA; rabbit anti-collagen I antibody, Abcam, USA; rat anti-LAMP2 antibody, Abcam, USA; mouse anti-GFAP antibody, Sigma, USA; rabbit anti-C3 antibody, Abcam, USA; rabbit anti-NeuN antibody, Millipore, USA; mouse anti-cleaved caspase-3 antibody, Cell Signaling Technology, USA) and then incubated with secondary antibodies. Apoptosis was detected using a transferase-mediated deoxyuridine triphosphate-biotin nick end labeling Kit (TUNEL Apoptosis Detection Kit, Roche, Switzerland). Slices were embedded using Fluoroshield^™^ with DAPI (Sigma, USA). Images were acquired using a Nikon fluorescence microscope (Nikon, Japan) or a confocal microscope (Leica, Germany).

### Statistical analysis

All data were analyzed by an investigator blinded to the group allocation. Image J (National Institutes of Health, USA) was used to analyze the immunohistochemical and western blotting results. Repeated measures two-way analysis of variance followed by Tukey’s post hoc multiple comparison tests were performed using GraphPad prsim 8.0. All data are presented as means ± standard deviations; *P* < 0.05 is considered statistically significant.

## Supplementary information

Fig.S1

Fig. S2

Fig. S3

Fig. S4

SUPPLEMENTAL MATERIAL
