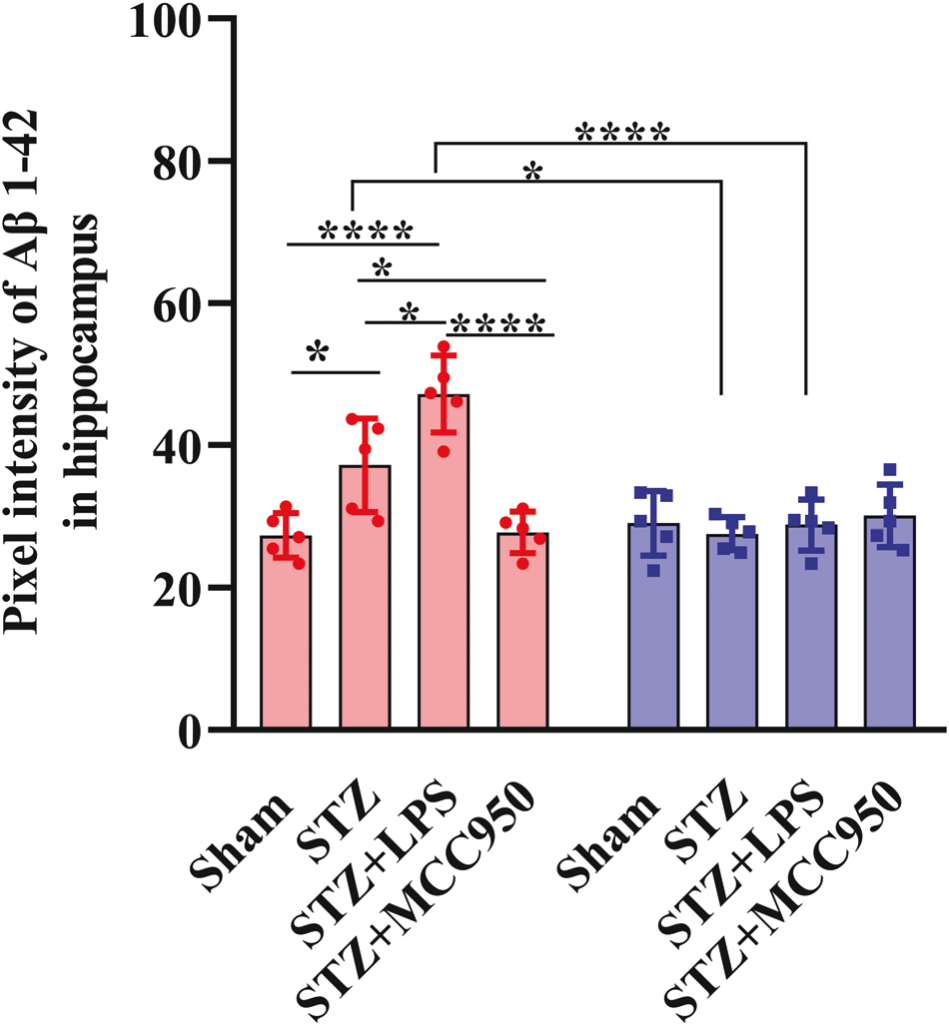# Correction to: NLRP3-dependent microglial training impaired the clearance of amyloid-beta and aggravated the cognitive decline in Alzheimer’s disease

**DOI:** 10.1038/s41419-022-04951-1

**Published:** 2022-05-23

**Authors:** Xiao-fei He, Jing-hui Xu, Ge Li, Ming-yue Li, Li-li Li, Zhong Pei, Li-ying Zhang, Xi-quan Hu

**Affiliations:** 1 Department of Rehabilitation Medicine, The Third Affiliated Hospital, Sun Yat-sen University, 510630 Guangzhou, Guangdong China; 2 The Eighth Affiliated Hospital, Sun Yat-sen University, 518000 Shenzhen, Guangdong China; 3 Guangdong Provincial Key Laboratory of Laboratory Animals, Guangdong Laboratory Animals Monitoring Institute, 510663 Guangzhou, Guangdong China; 4 Department of Neurology, National Key Clinical Department and Key Discipline of Neurology, Guangdong Key Laboratory for Diagnosis and Treatment of Major Neurological Diseases, The First Affiliated Hospital, Sun Yat-sen University, 510080 Guangzhou, Guangdong China

**Keywords:** Cellular neuroscience, Alzheimer's disease, Inflammasome

Correction to: *Cell Death and Disease* 10.1038/s41419-020-03072-x, published online 13 October 2020

The original version of this article unfortunately contained a mistake. The authors state the following: After publication of the article, we recently identified after re-reviewing this manuscript that the image from the “Pixel intensity of Aβ 1–40 in hippocampus” in figure 4. B showed overlap with the comparison histogram in figure 4.D due to mistaken figure production. Actually, we aimed to showing the histogram image from “Pixel intensity of Aβ 1-42 in hippocampus” in figure 4. B. The serious mistake was caused by unintentionally and carelessly mistaking the “Comparisons of Aβ 1–40 fragments in hippocampus” instead of “Comparisons of Aβ 1–42 fragments in hippocampus” during figure production. In fact, the careless mistakes do not affect the findings or conclusions of the article. The authors apologize for the error. The corrected figure can be found below. The original article has been corrected.