# Supplementary figures and images for: NLRP3-dependent microglial training impaired the clearance of amyloid-beta and aggravated the cognitive decline in Alzheimer’s disease
Source: Cell Death Dis. 2020 Oct 13;11(10):849. doi: 10.1038/s41419-020-03072-x (PMC7555905; doi:10.1038/s41419-020-03072-x)

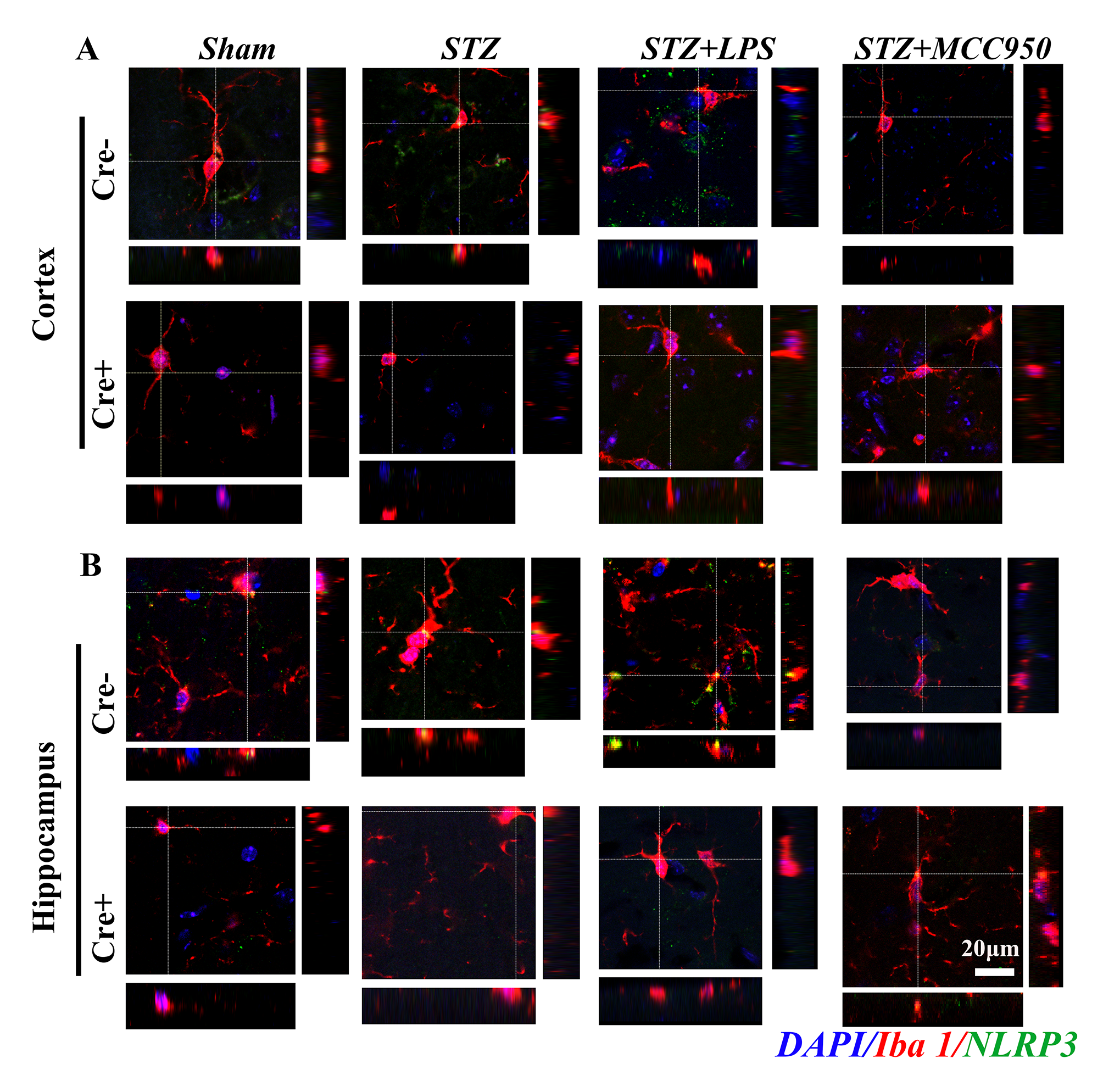

Supplement: Supplementary file 1 — Fig.S1 [file 41419_2020_3072_MOESM1_ESM.tif]

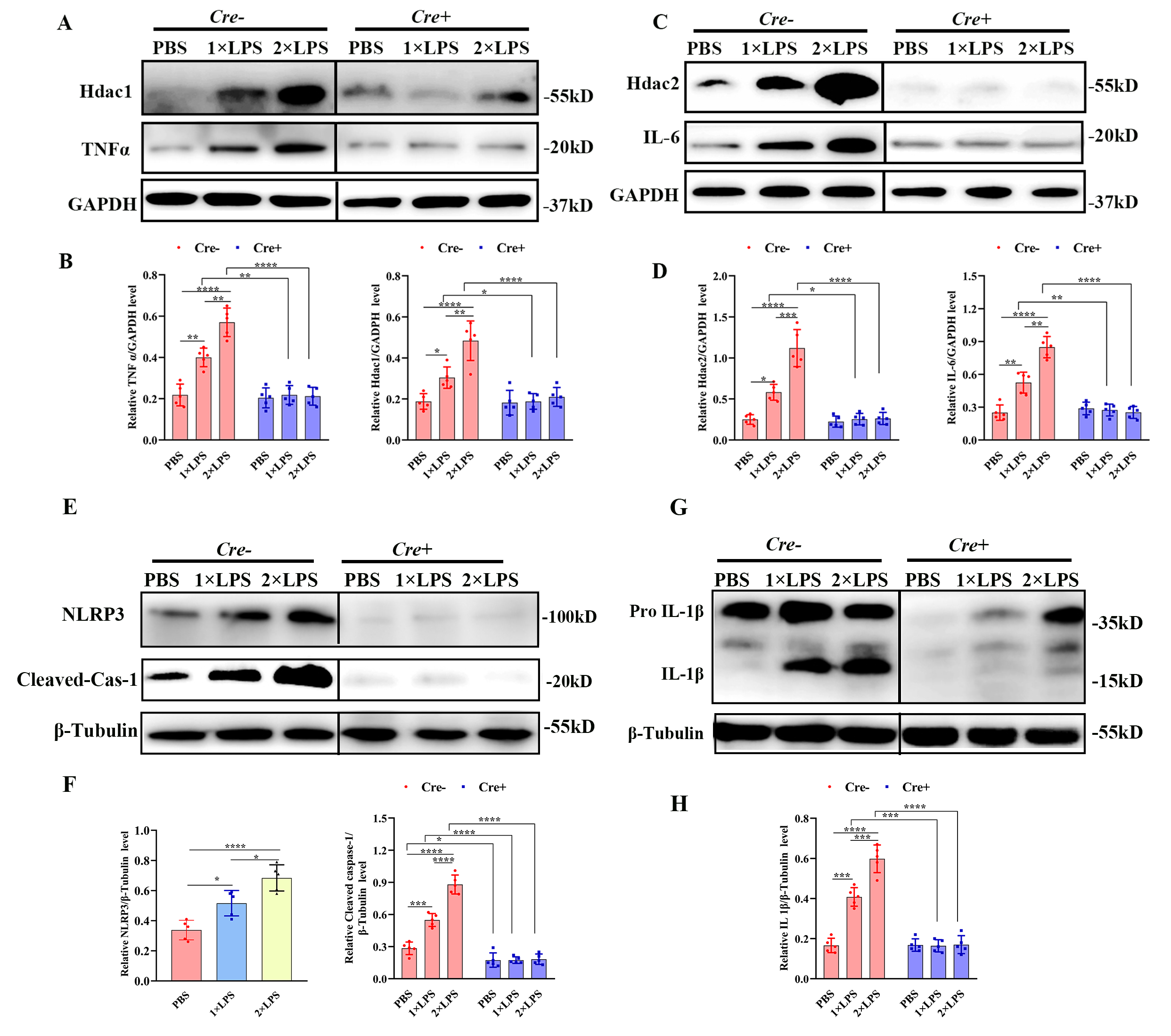

Supplement: Supplementary file 2 — Fig. S2 [file 41419_2020_3072_MOESM2_ESM.tif]

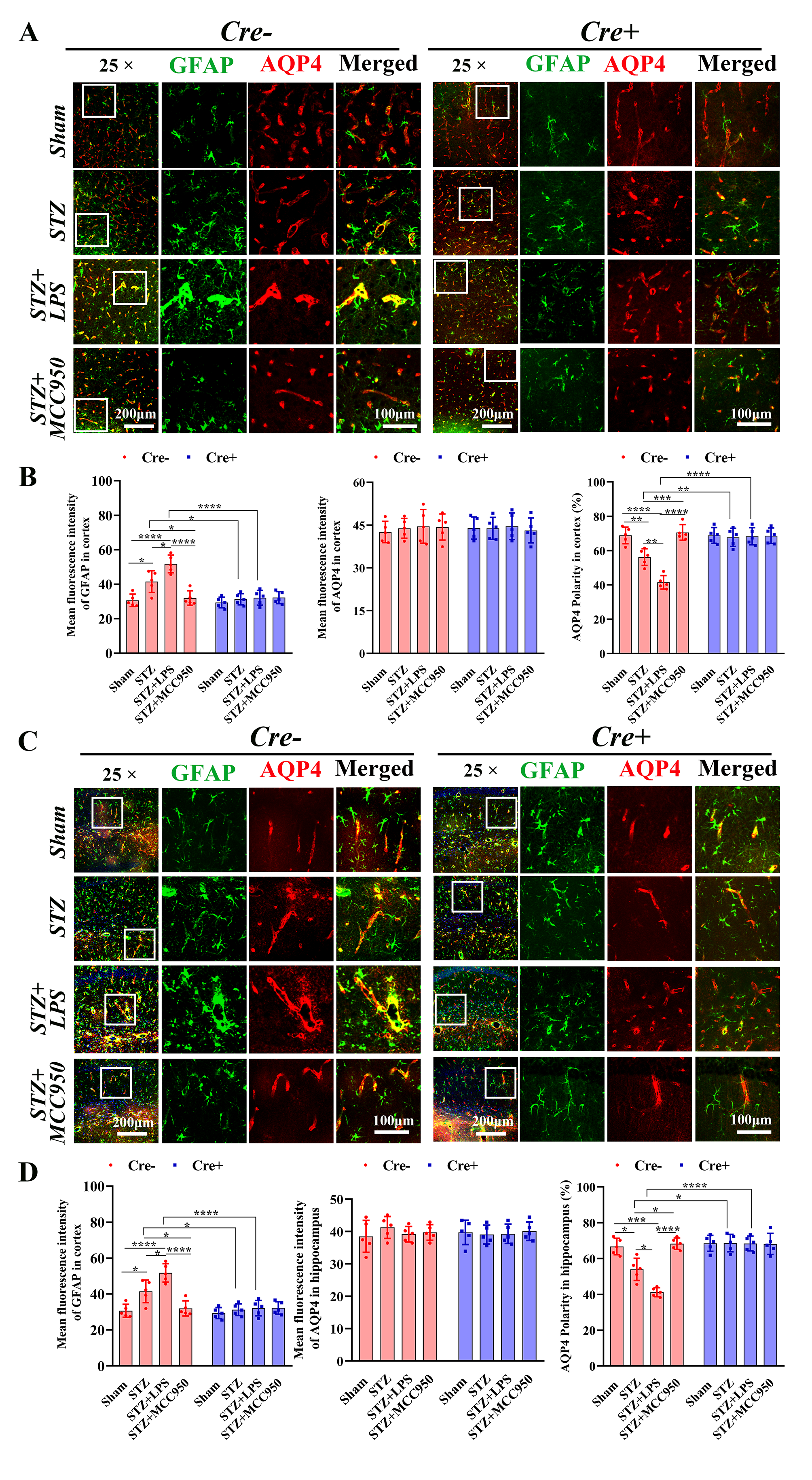

Supplement: Supplementary file 3 — Fig. S3 [file 41419_2020_3072_MOESM3_ESM.tif]

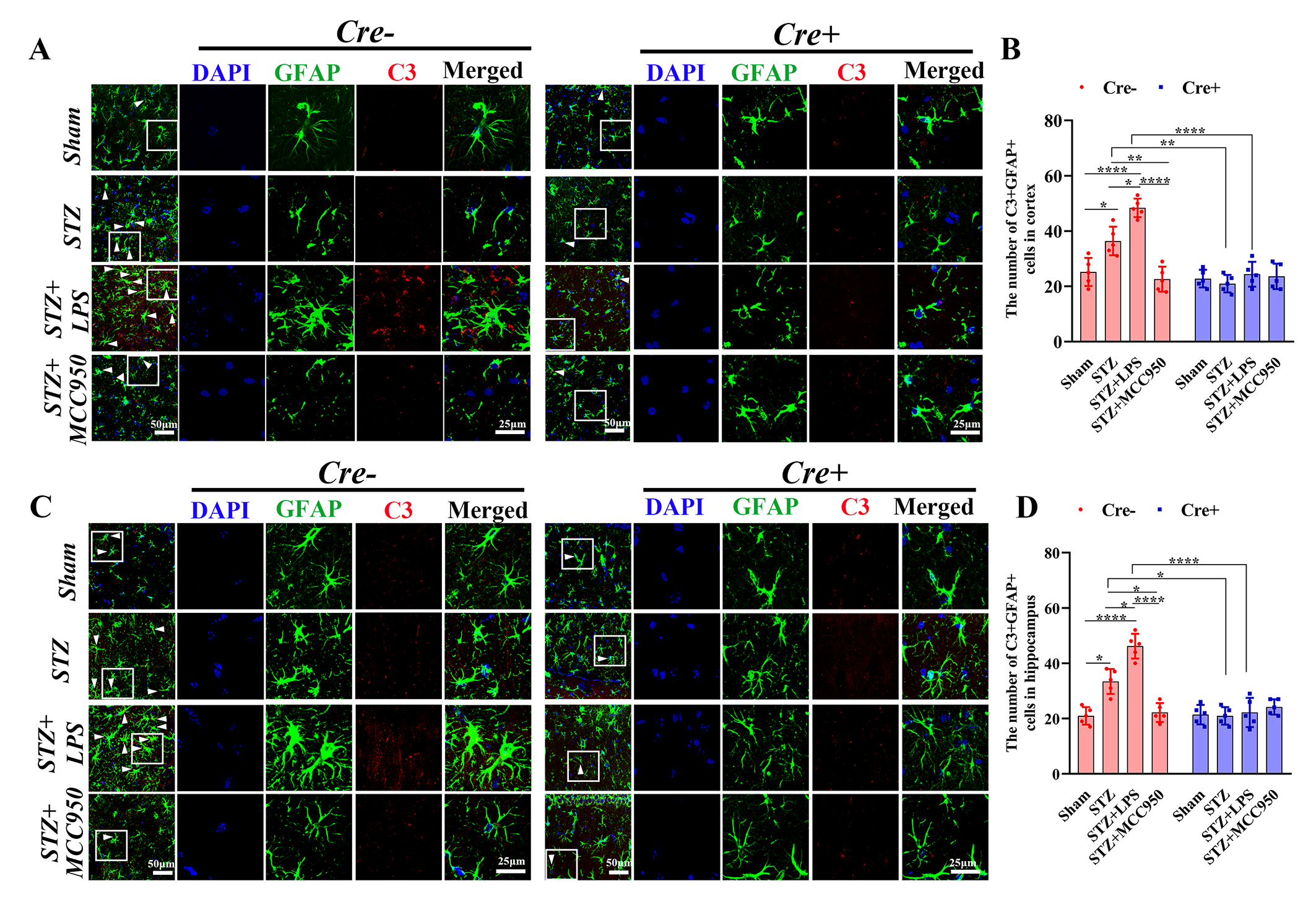

Supplement: Supplementary file 4 — Fig. S4 [file 41419_2020_3072_MOESM4_ESM.tif]
