# Supplementary material for: NLRP3-dependent microglial training impaired the clearance of amyloid-beta and aggravated the cognitive decline in Alzheimer’s disease
Source: Cell Death Dis. 2020 Oct 13;11(10):849. doi: 10.1038/s41419-020-03072-x (PMC7555905; doi:10.1038/s41419-020-03072-x)
Supplement: Supplementary file 5 — SUPPLEMENTAL MATERIAL [file 41419_2020_3072_MOESM5_ESM.docx]

Fig. S1: Three-dimensional images of NLRP3 and Iba1 positive cells in Cre- and Cre+ mice. A. Three-dimensional images of NLRP3 and Iba1 positive cell in the cortex (63 × oil immersion objective, zoomed in 3). B. Three-dimensional images of NLRP3 and Iba1 positive cell in the hippocampus (63 × oil immersion objective, zoomed in 3).

Fig. S2: Western blotting analysis of inflammatory cytokine and immune training associated proteins following i.p. injection of PBS, 1× and 2× LPS. A. Chemiluminescence imaging of Hdac1, TNF-α and GAPDH. B. Comparisons of Hdac1/GAPDH and TNF-α/GAPDH ratios among groups with PBS, 1 × LPS, and 2 × LPS injection in Cre- and Cre+ mice. C. Chemiluminescence imaging of Hdac2, IL-6, and GAPDH. D. Comparisons of Hdac2/GAPDH and IL 6/GAPDH ratios among groups with PBS, 1 × LPS, and 2 × LPS injection in Cre- and Cre+ mice. E. Chemiluminescence imaging of NLRP3, caspase-1, and β-tubulin. F. Comparisons of NLRP3/β-tubulin and cleaved caspase 1/β-tubulin ratios. G. Chemiluminescence imaging of IL-1β and /β-tubulin. H. Comparisons of IL-1β/β-tubulin ratios. Each dataset is expressed as mean ± SD. ^*^*P* ≤ 0.05; ^**^*P* ≤ 0.01; ^***^*P* ≤ 0.001; ^****^*P* ≤ 0.0001. n=6 mice.

Fig. S3: Histological analysis of astrocytic activation and astrocytic AQP4 polarity for the cortex and hippocampus in Cre- and Cre+ mice. A. Representative confocal images of GFAP+ astrocytes and AQP4 in the cortex. Left panel: Images under 25 × water immersion objective, white boxes indicate the area which was zoomed in 3 shown in right panels. Right panel: Images zoomed in 3 of areas indicated in left panel. B. Comparisons of the GFAP+ astrocyte intensity, AQP4 intensity, and AQP4 polarity in the cortex. C. Representative confocal images of GFAP+ astrocytes and AQP4 in the hippocampus. Left panel: Images under 25 × water immersion objective, white boxes indicate the area which was zoomed in 3 shown in right panels. Right panel: Images zoomed in 3 of areas indicated in left panel. D. Comparisons of the GFAP+ astrocyte intensity, AQP4 intensity, and AQP4 polarity in the hippocampus. Each dataset is expressed as mean ± SD. ^*^*P* ≤ 0.05; ^**^*P* ≤ 0.01; ^***^*P* ≤ 0.001; ^****^*P* ≤ 0.0001. n=6 mice.

Fig. S4: Histological analysis of astrocytic activation and phenotype in the cortex and hippocampus. A. Representative confocal images showing the GFAP+ positive astrocytes in the cortex in Cre- and Cre+ mice. Left panel: 63 × oil immersion objective, white boxes indicate the areas zoomed in 3 shown in the right panel. White arrow indicates the co-localization of C3 and GFAP. Right panel: Representative pictures zoomed in 3 for the white boxes in the left panel. B. Comparisons of the C3+GFAP+ cells in the cortex. C. Representative confocal images showing the GFAP+ positive astrocytes in the hippocampus in Cre- and Cre+ mice. Left panel: 63 × oil immersion objective, white boxes indicate the areas zoomed in 3 shown in the right panel. White arrow indicates the co-localization of C3 and GFAP. Right panel: Representative pictures zoomed in 3 for the white boxes in the left panel. D. Comparisons of the C3+GFAP+ cells in hippocampus. Each dataset is expressed as mean ± SD. ^*^*P* ≤ 0.05; ^**^*P* ≤ 0.01; ^***^*P* ≤ 0.001; ^****^*P* ≤ 0.0001. n=6 mice.
